# Skeletal Cell Differentiation Is Enhanced by Atmospheric Dielectric Barrier Discharge Plasma Treatment

**DOI:** 10.1371/journal.pone.0082143

**Published:** 2013-12-12

**Authors:** Marla J. Steinbeck, Natalie Chernets, Jun Zhang, Deepa S. Kurpad, Gregory Fridman, Alexander Fridman, Theresa A. Freeman

**Affiliations:** 1 Department of Biomedical Engineering, Drexel University, Philadelphia, Pennsylvania, United States of America; 2 Department of Electrical Engineering, Drexel University, Philadelphia, Pennsylvania, United States of America; 3 Department of Orthopaedics, The Second Hospital of Jilin University, Chang Chun, Jilin, China; 4 Department of Orthopaedic Surgery, Thomas Jefferson University, Philadelphia, Pennsylvania, United States of America; 5 Department of Mechanical Engineering and Mechanics, Drexel University, Philadelphia, Pennsylvania, United States of America; Institute for Frontier Medical Sciences, Kyoto University, Japan

## Abstract

Enhancing chondrogenic and osteogenic differentiation is of paramount importance in providing effective regenerative therapies and improving the rate of fracture healing. This study investigated the potential of non-thermal atmospheric dielectric barrier discharge plasma (NT-plasma) to enhance chondrocyte and osteoblast proliferation and differentiation. Although the exact mechanism by which NT-plasma interacts with cells is undefined, it is known that during treatment the atmosphere is ionized generating extracellular reactive oxygen and nitrogen species (ROS and RNS) and an electric field. Appropriate NT-plasma conditions were determined using lactate-dehydrogenase release, flow cytometric live/dead assay, flow cytometric cell cycle analysis, and Western blots to evaluate DNA damage and mitochondrial integrity. We observed that specific NT-plasma conditions were required to prevent cell death, and that loss of pre-osteoblastic cell viability was dependent on intracellular ROS and RNS production. To further investigate the involvement of intracellular ROS, fluorescent intracellular dyes Mitosox (superoxide) and dihydrorhodamine (peroxide) were used to assess onset and duration after NT-plasma treatment. Both intracellular superoxide and peroxide were found to increase immediately post NT-plasma treatment. These increases were sustained for one hour but returned to control levels by 24 hr. Using the same treatment conditions, osteogenic differentiation by NT-plasma was assessed and compared to peroxide or osteogenic media containing β-glycerolphosphate. Although both NT-plasma and peroxide induced differentiation-specific gene expression, neither was as effective as the osteogenic media. However, treatment of cells with NT-plasma after 24 hr in osteogenic or chondrogenic media significantly enhanced differentiation as compared to differentiation media alone. The results of this study show that NT-plasma can selectively initiate and amplify ROS signaling to enhance differentiation, and suggest this technology could be used to enhance bone fusion and improve healing after skeletal injury.

## Introduction

The goal of this investigation was to examine the effect of non-thermal (NT)-plasma on skeletal cell differentiation. Medical use of plasma technology is most commonly associated with thermal plasmas, such as the plasma knife used in surgery to cut and simultaneously cauterize vessels as a result of the high temperature generated by plasma [1]. Advancements in atmospheric pressure plasma systems led to the development of a novel NT dielectric barrier discharge plasma with a discharge sufficiently uniform and cold to safely apply to living cells and tissues [2]–[6]. The NT-plasma discharge is generated by applying a high voltage, time-varying waveform between a dielectric covered electrode and the biological target [7], [8]. To prevent high temperature build-up and transition to arc, high voltage current is alternated between the two electrodes, one of which is covered with a dielectric. Within the NT-plasma discharge, the molecules present in air (O_2_, N_2_, H_2_O, CO_2_, etc) are ionized resulting in the direct formation of numerous reactive oxygen species (ROS) and reactive nitrogen species (RNS) [8]–[10].

Most biomedical studies on the effect of NT-plasma have focused on the bacteriostatic and bactericidal properties of this new technology [11]–[13]. Recently, it was reported that NT-plasma exposure promoted endothelial cell proliferation, which was abrogated by fibroblast growth factor (FGF)-2 neutralizing antibody [5]. Proliferation and FGF-2 release were blocked by N-acetyl-cysteine (NAC), which prevented changes in intracellular redox. Mechanistically, these studies directly link NT-plasma effects to ROS or RNS generation.

ROS and RNS are known to directly activate multiple proteins involved in signaling pathways that regulate cell function. ROS-responsive MAP kinases are known to control a wide range of cellular processes including: cellular differentiation, cell cycle control, cytokine and growth factor signaling, survival, hypertrophy and/or apoptosis [14]–[17]. For example, the Map5kinase Apoptosis signal-regulating kinase 1 (ASK1), is particularly sensitive to ROS as its activity is tightly regulated by four ROS sensitive proteins thioredoxin, glutaredoxin, Akt and 14-3-3 [17]–[21]. ROS activated ASK1 phosphorylates and activates both p38 and jnk kinases, which play key roles in cellular differentiation [22], [23] as well as the regulation of apoptosis [24]. Activation of ASK1, p38 and/or jnk promotes the differentiation of several cell lineages including chondrocytes [25]–[27], osteoblasts, neuronal [28], myoblasts [29] and keratinocytes [14], [23], [27].

In the case of mesenchymal cell differentiation into osteoblast lineages there is precedence for ROS stimulation to both direct and enhance this process [30]–[32]. Similarly, enhanced chondrogenesis has also been associated with ROS stimulation [27] and the occurrence of oxidative spikes as a driving force for transitional stages throughout developmental and regenerative processes is a commonly observed phenomena. The possibility of using NT-plasma technology to artificially trigger these transitions and direct these processes is intriguing. Herein, we show that specific exposure conditions to NT-plasma promote intracellular ROS production and skeletal cell differentiation.

## Materials and Methods

### Cell Culture

The pre-osteocytic cell line MLO-A5 was obtained from Dr. Linda Bonewald and cultured using the method described previously [33]. To induce maturation for qRT-PCR experiments MLO-A5, cells were placed in osteogenic differentiation media (Dulbecco’s Modified Eagle’s Medium (DMEM) (Cellgro, Fisher Scientific, USA) with 10% Fetal Bovine Serum (FBS) (Invitrogen, Life Science Technologies, USA), and supplemented with 100 units/ml penicillin, 100 µg/ml streptomycin (Cellgro, Fisher Scientific, USA), 0.5 mM beta glycero-phosphate and 100 µg/ml ascorbic acid (Sigma-Aldrich, St. Louis, MO) and incubated in 5% CO_2_ at 37°C with media changes every 2 days. N1511 mouse chondrocytes were obtained from Dr. Motomi Enomoto-Iwamoto. The cells were maintained in culture using the method described previously [34], [35]. Briefly, the cells were plated at a concentration of 50,000 cells/ml in/−MEM containing 10% FBS, 0.2% L-glutamine, and penicillin/streptomycin. To induce maturation, after 24 h, the adherent cells were treated with 200 ng/ml recombinant BMP2 (Alpha Diagnostic Intl., San Antonio, TX). Assessment of the maturation was performed at day 5 by measuring alkaline phosphatase activity and the expression of chondrogenic markers.

### Power Measurement for Calibration of Plasma Device

Tektronix DPO4104B (1 GHz bandwidth, 5 GS/sec) digital oscilloscope with Tektronix P6015A high voltage divider (1000∶1) and Ion Physics Corp CM-10-L current probe (1 V/1 A, 5 kA max) was used to record voltage and current waves at maximum sampling rate. Instant values for both were multiplied and resulting instant power was then integrated over the entire cycle. Ten measurements were taken for statistics analysis.

### Non-Thermal Plasma Treatment

NT-plasma was generated by applying alternating polarity pulsed voltage of 20 kV magnitude (peak to peak), a 10 µs pulse width, at a frequency between 50 to 3500 Hertz (Hz), with a rise time of 5 V/ns between the quartz-insulated high voltage electrode and the sample undergoing treatment 2 mm from the electrode. General electrical schematics and description of the power supply (Plasma Power, LLC, Philadelphia USA) were as reported by the authors [4]. A 32 mm (6-well plate) electrode was used, which was composed of an inner core of copper surrounded by an outer insulating shell of acrylic, and a 0.5 mm quartz disc plasma-generating surface covered with a 0.01inch sheet of polyether ether ketone. Just before treatment, culture media was removed by pipet from the dish and replaced immediately following treatment.

### Cell Viability and Cell Cycle Analysis

Cell viability was measured by live/dead flow cytometry in cells stained with propidium iodide (PI; Sigma), an indicator of cell death. PI was added immediately before analysis on the MACSQuant VYB flow cytometer (Miltenyi Biotec, Auburn, CA). The number of PI stained cells was counted and the data was analyzed with MACSQuant software (Miltenyi Biotec, Auburn, CA).

Lactate dehydrogenase (LDH) (Cayman Chemical, Ann Arbor, MI) assay was performed at 24 hr as per manufacturer’s instructions, and the % cytotoxicity (LDH release) was calculated from the standard curve and TritonX-100 positive control. Intensity measurements were performed using a Tecan Infinite 1000 plate reader (Tecan, Research Triangle, NC). H_2_O_2_ (100 µM) (Life Technologies, Grand Island, NY) or 1-oxyl-2,2,6,6-tetramethyl-4-hydroxypiperidine (***TEMPOL***; ***6*** ***mM***) (Sigma, St. Louis, MO) was added to the medium for one hour before NT-plasma treatment. The media was replaced after 3 hr without H_2_O_2_ or TEMPOL.

To determine cell cycle kinetics, 24 hr after NT-plasma treatment cells were prepared for flow cytometric cell cycle analysis on the MACSQuant VYB (Miltenyi Biotec, Auburn, CA), following the Miltenyi cell cycle analysis protocol. Briefly, cells were collected in sample buffer (Ca^+2^ and Mg^+2^ free PBS, with 1 g/L glucose), washed and placed in 70% ethanol overnight at 4°C. After re-suspension in sample buffer, the propidium iodide (PI (1 ug/ml); Sigma, St. Louis, MO) was added and the stained cells were analyzed with MACSQuant software (Miltenyi Biotec, Auburn, CA). This procedure was repeated in quadruplicate on 3 separate days.

### Western Blot Analysis for DNA and Mitochondrial Damage

Protein from NT-plasma treated cells was collected 24 hr after treatment. Cells were isolated with MPER®, 5 M NaCl, complete protease inhibitor cocktail, DTT, 100 µM NaF, and 100 µM Na_3_VO_4_ and quantified using the Bradford assay (Pierce Biotechnology Thermol Scientific, Rockford IL). Protein (40 µg) was then loaded onto 10% Tris polyacrylamide gels. Samples were transferred to PVDF membranes and subsequently blocked with ECL Blocking agent (RPN 2125, GE Health Life Sciences, Cleveland, OH) (2.0% TBS-T +2.0 g dried milk). Blots were treated overnight with antibodies to either H2Ax (Millipore, Billerica, MA) or Cytochrome C (BD Biosciences, Franklin Lakes, NJ) at a 1∶500 dilution in 2% blocking reagent. The blots were washed three times in TBST and species specific HRP conjugated secondary antibodies (Santa Cruz Biotechnology, Dallas, TX) were applied for 1 hr at room temperature. After three washes in TBST, the membrane was detected with Western Bright Quantum kit (Advansta, Menlo Park, CA) for 5 min at 25°C. Photographs of the membranes were taken in a GE Image quant LAS 4000 dark box with a GE Luminescent Image Analyzer LAS-4000 (GE Health Life Sciences, Cleveland, OH) and digital images were collected using the Image quant TL 1D gel analysis software (GE Health Life Sciences, Cleveland,OH).

### ROS Measurements

Mitosox and dihydrorhodamine (DHR) dyes (Invitrogen, Carlsbad, CA) were used to detect the presence of intracellular superoxide anion and hydrogen peroxide, respectively. NT-plasma or sham treatments were performed on MLO-A5 cells plated overnight in 6 well plates and fluorescent intensity was measured using a Tecan Infinite 1000 plate reader (Tecan, Research Triangle, NC). Inhibitors (NAC, 200 µM and TEMPOL, 6 mM (Sigma, St. Louis, MO) were added to the medium for one hour before NT-plasma treatment and replaced 3 hr after with regular media.

### Real Time-PCR for Differentiation Markers and Comparative Analyses

Confluent cells in 6 well dishes were either treated with differentiation media (β-GP), H_2_O_2_ (100 µM) or treated with 1000 Hz NT-plasma for 10 sec and harvested after 24 hr. Alternatively, confluent cells from 6 well dishes were placed in differentiation media (MLO-A5 with β-GP or N1511 with BMP) 24 hr before NT-plasma or sham-plasma control treatment and harvested after 24 hr or 56 hr. All harvested cells were washed with DEPC water before total RNA was isolated using the QiagenRNeasy® Mini kit (Qiagen, Valencia, CA). RNA yield was determined spectrophotometrically and integrity confirmed by gel electrophoresis. RNA was reverse-transcribed and then amplified using the Superscript™ One-Step RT-PCR with Platinum Taq® (Invitrogen, Carlsbad, CA) kit. PCR products were analyzed by 1.0% agarose gel electrophoresis. Template cDNA and gene specific primers were added to Fast SYBR Green master mix (Applied Biosystems, Carlsbad, CA) and mRNA expression was quantified using the MyIQ Real-Time PCR System (BioRad, Hercules, CA). The expression level of the housekeeping gene, β-actin was used to normalize the data presented. Melting curves were analyzed to verify the specificity of the RT-PCR reaction and the absence of primer dimer formation. Each sample is analyzed in duplicate and included a template-free control. All the primers (Table 1) used were synthesized by Integrated DNA Technologies, Inc. (Coralville, IN).

**Table 1:** PCR primer information.

Primer Name	Strand	Primer Sequence
actin	FWD	gct aca gct cac cac cac a
actin	REV	251658240tct cca ggg agg aag agg a
Osteocalcin	FWD	gac aaa gcc ttc atg tcc aag c
Osteocalcin	REV	aaa gcc gag ctg cca gag ttt g
BMP2	FWD	aag aag ccg tgg agg aac tt
BMP2	REV	ttc ccg gaa gat ctg gag tt
BSP	FWD	agg gag gca gtg act ctt ca
BSP	REV	aca ccc gag agt gtg gaa ag
Runx2	FWD	gcc ggg aat gat gag aac ta
Runx2	REV	gga ccg tcc act gtc act tt
Fgf2	FWD	agc ggc tct act gca aga ac
Fgf2	REV	gcc gtc cat ctt cct tca ta
ALK PHOS	FWD	tat gtc tgg aac cgc act ga
ALK PHOS	REV	cca gca aga aga agc ctt tg
Col X	FWD	cgt gtc tgc ttt tac tgt ca
Col X	REV	acc tgg tca ttt tct gtg ag
MMP-13	FWD	atc ctg gcc acc ttc ttc tt
MMP-13	REV	ttt ctc gga gcc tgt caa ct
OSTERIX	FWD	act ggc tag gtg gtg gtc ag
OSTERIX	REV	ggt agg gag ctg ggt taa gg

### Statistical Analysis

Statistical analysis between groups was performed using a one-way ANOVA for normality and student’s t-test for continuous variables. A level of significance (α), or a p-value of less than 0.05, with a 95% confidence interval was determined. Representative data are presented as the mean ± SD of individual samples from 2 to 16 independent analyses, unless otherwise stated. In this work “n” indicates number of separate experimental trials, with repeats varying from 3 to 8, or more. For normally distributed data, differences between groups were determined by independent t-tests. For non-parametric data, Mann Whitney U tests were used to evaluate differences.

## Results

### Determining the Appropriate NT-Plasma Dose

**Figure 1:**
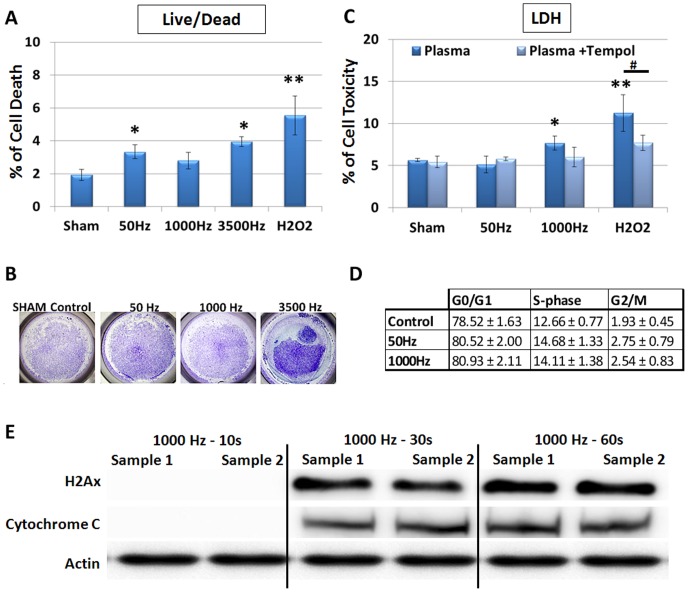
Direct effects of NT-plasma on cytotoxicty and cell proliferation. Osteoblast-like, MLO-A5 cells were treated with NT-plasma at frequencies of 50, 1000 and 3500 Hz for 10 seconds. (**A**) Cell death measured by nuclear PI incorporation at 24 hr showed increased cell death in response to frequency 50 Hz, 3500 Hz (p<0.01) and H_2_O_2_ (p<0.001) (n = 3). There was no significant cell death in response to frequency 1000 Hz. (**B**) Cell detachment was observed at 3500 Hz as shown by toluidine staining of cells after NT-plasma treatment. (**C**) Cell viability after NT-plasma was assessed by a lactate dehydrogenase (LDH) release assay. 1000 Hz and H_2_O_2_ both showed an increase in LDH release (p<0.01 and p<0.001). LDH release was reduced in the presence of TEMPOL for both H_2_O_2_ (p<0.01) and NT-plasma treatments (ns, n = 3). (**D**) No difference in cell cycle profile was observed between NT-plasma at 50 Hz or 1000 Hz as compared to sham control. (**E**) Western blots show no H2Ax or cytochrome c release in cells treated at 1000 Hz for 10 s as compared to the 30 and 60 sec NT-plasma treatments. Statistical significance was determined by the Mann-Whitney U test for non-parametric data; * or # (p<0.01) ** or ## (p<0.001.

To investigate the appropriate NT-plasma dose, MLO-A5 osteoblastic cells in 6 well plates were treated with NT-plasma for 10 sec at a voltage of 20 kV and pulse width of 10 µs), varying only the frequency at either 50, 1000, or 3500 Hertz (Hz). Controls included the NT-plasma electrode without power as a sham treatment, and H_2_O_2_ (100 µM) as a positive control for ROS. Cell death was determined 24 hr after NT-plasma treatment using a fluorescent flow cytometric live/dead assay. Less than 4% of the cells underwent cell death in response to NT-plasma treatment, although the 50 Hz (p<0.05) and 3500 Hz (p<0.05) treatments were significantly above control (Fig. 1A). H_2_O_2_ induced a slightly higher 5.6% increase in cell death (p<0.01). Results for the 3500 Hz treatment, were an under estimate of cell damage, as large areas of the culture plate were devoid of cells immediately after treatment (Fig. 1B). Due to cell detachment this setting was not used in subsequent assays.

To further assess the 50 and 1000 Hz dose rates, MLO-A5 cell cytotoxicity was measured using lactate dehydrogenase (LDH) (Fig. 1C). At 24 h LDH release in response to 50 Hz was 5.1% (not significant; ns) and 1000 Hz was 7.7% (p<0.01) above NT-plasma sham control. Both treatments were less than H_2_O_2_, which induced an 11.2% increase in cell death (p<0.01). To prevent changes in intracellular oxidant levels, TEMPOL (6 mM) a membrane permeable scavenger of both ROS and RNS, was added 1 hr before NT-plasma or H_2_O_2_ treatment [36], [37]. TEMPOL reduced the release of LDH in response to 1000 Hz NT-plasma (ns) and significantly decreased LDH release in response to H_2_O_2_ (p<0.01), indicating ROS/RNS are being generated in response to NT-plasma and cause cell damage.

To determine whether NT-plasma treatment affected cell proliferation (increased S phase) or caused DNA damage (blockage in G2), flow cytometric analysis of cell cycle phase was determined 24 hours after treatment of MLO-A5 cells with 50 and 1000 Hz NT-plasma or H_2_O_2_ (Fig. 1D). No significant differences were observed in the G1, S or G2/M phase after any treatment, indicating no increase in cell proliferation or blockage of the cell cycle due to DNA damage.

Additionally, Western blot analysis was performed to assess damage to DNA or mitochondrial membranes using antibodies to the histone2A variant (H2AX) and cytoplasmic cytochrome c, respectively (Fig. 1E). The 1000 Hz NT-plasma treatment dose was achieved by varying the time to include 10 sec (4.08 Joules (J)/cm^2^), 30 sec (12.24 J/cm^2^) and 60 sec (24.48 J/cm^2^) dose. The 10 sec dose did not induce DNA or mitochondrial damage within 1 hr after treatment, but damage after both the 30 and 60 sec treatments was observed.

Taken together the results show that NT-plasma treatment at 1000 Hz for 10 sec did not significantly reduce cell viability, alter the cell cycle or cause DNA or mitochondrial damage in MLO-A5 cells. Based on these findings the 1000 Hz, 10 sec NT-plasma treatment was chosen as the NT-plasma optimal treatment.

### Production of Intracellular ROS in Response to NT-Plasma

**Figure 2:**
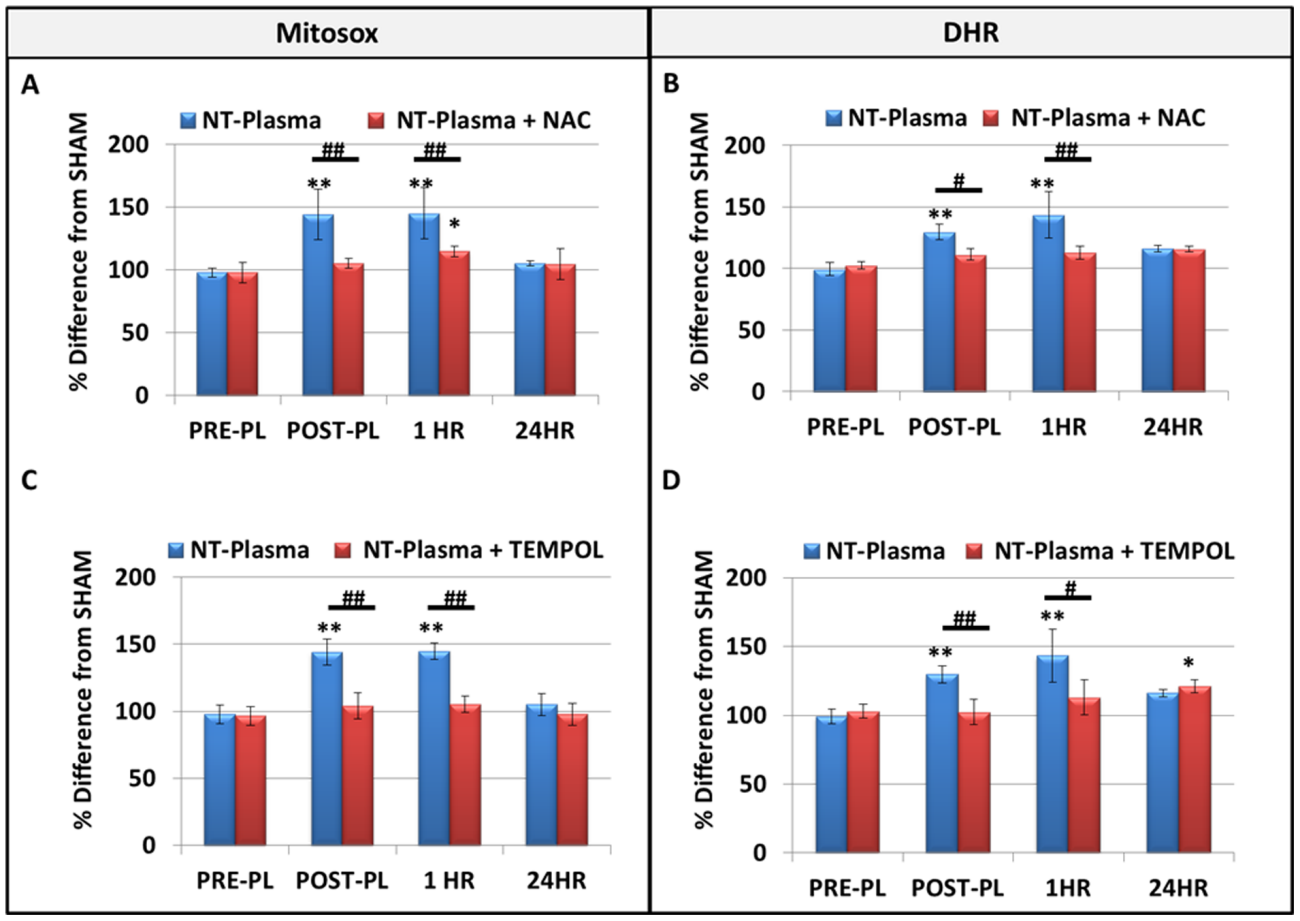
Direct effects of NT-plasma on intracellular ROS production. (**A and B**) Intracellular O_2_^−**.**^ and H_2_O_2_ levels were measured using fluorescent indicators MitoSox (n = 3) and dihydrorhodamine (DHR), (n = 3) respectively. Immediately post-treatment (POST-PL) and at 1 hr, 1000 Hz NT-plasma treated cells generated significantly increased amounts of O_2_^−**.**^ and H_2_O_2_ (p<0.001) as compared to sham control or pre-treatment levels (PRE-PL). Amounts of both ROS were decreased by 24 hr. NAC and TEMPOL quenched the ROS increase POST-PL and at 1 hr (p<0.01–0.001). However, H_2_O_2_ levels in the TEMPOL (p<0.01) inhibitor group were significantly increased above control at 24 hr, presumably due to removal of baseline NO**^.^** The results are expressed as the mean ± standard deviation. Statistical significance was determined by the Mann-Whitney U test for non-parametric data; * or # (p<0.01) ** or ## (p<0.001.

To evaluate intracellular reactive species levels in response to NT-plasma at 1000 Hz, 10 sec, mitochondrial superoxide anion (O_2_^−**.**^) and cytosolic/mitochondrial H_2_O_2_ levels were measured using fluorescent indicators MitoSox and dihydrorhodamine (DHR), respectively. Measurements were done immediately post-treatment (POST-PL), at 1 hr and 24 hr after NT-plasma and results were compared to sham controls (pre-plasma, PRE-PL). Immediately post-treatment and at 1 hr, NT-plasma treated cells showed significantly increased intracellular O_2_^−**.**^ (Fig. 2A) and H_2_O_2_ generation (p<0.001) (Fig. 2B) as compared to PRE-PL. Levels of both oxidants returned to PRE-PL levels by 24 hr. The immediate increase in H_2_O_2_ may be due to ROS produced by NT-plasma but the extended detection indicates ROS are being actively produced in response to NT-plasma.

To confirm the production of ROS in response to NT-plasma treatment, MLO-A5 cells were pretreated for 1 hr with either NAC (200 µM), to maintain intracellular redox balance and interfere with ROS production [38] or TEMPOL (6 mM), a scavenger of ROS as well as RNS. After pre-incubation with the inhibitor, the cells were subjected to NT-plasma treatment at 1000 Hz for 10 sec. NAC significantly inhibited the POST-PL and 1 hr NT-Plasma increase in both O_2_^−.^ (Fig. 2A) and H_2_O_2_ (Fig. 2B). TEMPOL also significantly inhibited the POST-PL and 1 hr increase in O_2_^−.^ (Fig. 2C), and it inhibited the POST-PL increase in H_2_O_2_ (Fig. 2D). To lesser extent TEMPOL inhibited the 1 hr increase in H_2_O_2_ in response to NT-Plasma, and at 24 hr H_2_O_2_ production was increased (p<0.01). One possible explanation for the decreased effectiveness of TEMPOL after 1 hr and 24 hr after NT-plasma/TEMPOL treatment is that removal of NO**^.^** prevents its reaction with O_2_^−**.**^ and the production of peroxynitrite (ONOO-). Thus, by scavenging NO**^.^** TEMPOL allows H_2_O_2_ to be generated from O_2_^−**.**^, rather than ONOO-, indicating some NO**^.^** is being generated although at significantly lower amounts compared to H_2_O_2_.

### NT-plasma Differentiation of Osteoblasts and Chondrocytes in vitro

**Figure 3:**
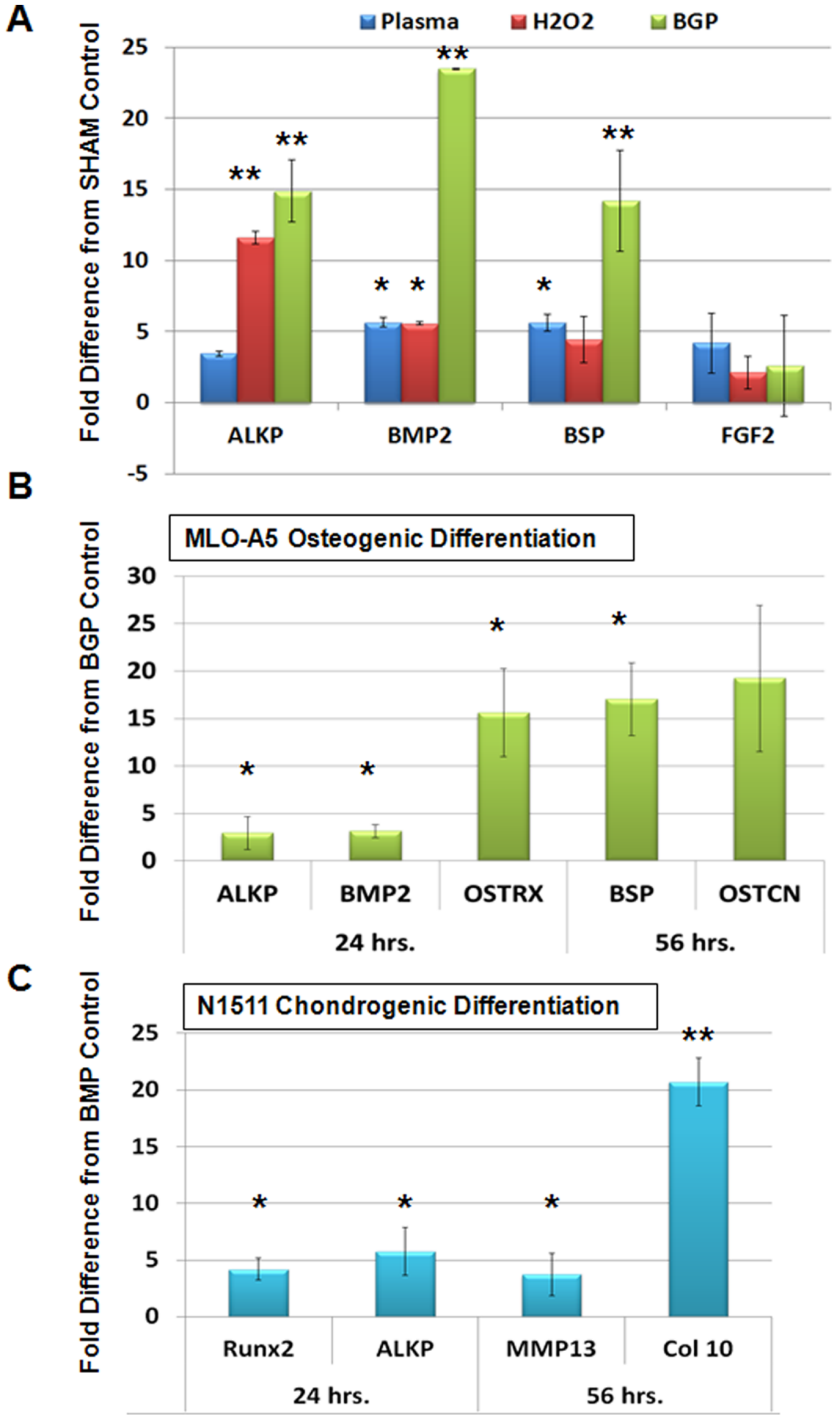
NT-plasma induced osteogenic differentiation using qPCR markers. (**A**) Fold increases in the expression of alkaline phosphatase (ALKP), bone morphogenetic protein 2 (BMP2), bone sialoprotein (BSP) and fibroblast growth factor-2 (FGF-2) in response to NT-plasma treatment, H_2_O_2_ or β-glycerol phosphate (βGP) normalized to sham treated control cells after 24 hr. ALKP was upregulated 3- (NS) and 11-fold (p<0.01), respectively, and BMP2 and BSP were increased 5 fold (p<0.05). βGP increased expression 14–23 fold (p<0.01). FGF-2 expression was not affected by NT-plasma, H_2_O_2_ or βGP. (**B**) After a 24 hr incubation in βGP NT-plasma was applied. 24 hr later there was a 2–7 fold increase in the induction of BMP2, ALKP (p<0.05) and Osterix was increased 15 fold (p<0.01) compared to βGP treatment alone. At 56 hr, BSP and osteocalcin (OSTCN) were increased 17–24 fold above βGP-treated control (p<0.05). The results are expressed as the mean ± standard deviation (n = 2). Statistical significance was determined by the Wilcoxin Mann-Whitney test for non-parametric data; * (p<0.05), ** (p<0.01).

To determine if NT-plasma treatment influenced the differentiation of MLO-A5 cells, alkaline phosphatase (ALKP), bone morphogenetic protein 2 (BMP2), bone sialoprotein (BSP) and fibroblast growth factor-2 (FGF-2) expression was evaluated by qPCR (Fig. 3). We compared differentiation media containing β-glycerophosphate (βGP) to either NT-plasma or H_2_O_2_ treatment; used to evaluate the effects of redox. Both NT-plasma and H_2_O_2_ increased the expression of osteogenic genes above controls, which were undifferentiated cells sham treated with NT-plasma (Fig. 3A). ALKP expression was upregulated 3 (ns) and 11 (p<0.01) fold, respectively, and BMP2 and BSP were increased 5 fold by 24 hr (p<0.05). However, compared to the 14–23 fold increases induced by βGP alone (p<0.01), these changes were relatively small. FGF-2 expression, which is increased in endothelial cells in response to NT-plasma [5], was not affected in MLO-A5 cells by NT-plasma, H_2_O_2_ or βGP. These results indicate NT-plasma does not significantly promote osteoblast differentiation when compared to βGP, although it induces some differentiation specific protein expression.

As NT-plasma treatment evoked only a weak induction of osteogenic gene expression, a potential synergism between βGP and NT-plasma was investigated. NT-plasma treatment applied 24 hr after incubation in βGP, induced a 2–7 fold increase in the expression levels of osteogenic markers BMP2 and ALKP above βGP alone (p<0.05) (Fig. 3B). The expression of osterix (ostrx), a key transcription factor for osteogenesis, was increased 15 fold (p<0.01). At 56 hr after NT-plasma treatment, the late osteoblast markers, BSP and osteocalcin (OSTCN) were increased 17–24 fold above βGP-treated control (p<0.05).

To determine if the differentiation effect of NT-plasma was osteoblast specific, the N1511 chondrocyte cell line was subjected to the same NT-plasma treatment in the presence of BMP2 (200 ng/ml), a known inducer of chondrocyte differentiation [34]. 24 hr after treatment, chondrocyte differentiation markers Runx2, ALKP were increased 3–6-fold above BMP-treated controls (p<0.05) (Fig. 3C). By 56 hr, collagen type X (Col X) and another late marker, matrix metalloprotease 13 (MMP13) were both increased 20 (p<0.01) and 4-fold (p<0.05), respectively above BMP-treated control.

We conclude from these studies that NT-plasma alone was not sufficient to initiate significant changes in cell differentiation. However, once differentiation had been initiated, NT-plasma enhanced osteogenesis and chondrogenesis at both early and late time points.

## Discussion

This study investigated the potential of NT-plasma to enhance both chondrocyte and osteoblast differentiation. Initially, we determined the specific NT-plasma condition required to maintain cell viability and showed both an immediate and long-term increase in intracellular ROS and RNS in response to NT-plasma. Furthermore, we demonstrate that NT-plasma enhanced the expression of differentiation specific genes by MLO-A5 in media without differentiation factors and in differentiation media, In media alone, the levels of expression were equal to those induced by H_2_O_2_, but not as effective as differentiation medium. However, when NT-plasma was applied 24 hr after the cells were placed in differentiation media a synergistic enhancement of chondrogenic and osteogenic differentiation was observed.

The NT-plasma generated ROS and RNS at the cell/environment interface initiates an immediate intracellular oxidative response (Fig. 2). It is interesting to speculate that this reaction may mimic naturally occurring ROS-associated cell signaling known to be associated with stem cell differentiation, cell fate decisions and regeneration. Within the stem cell pool, quiescence and pluripotency is maintained by the repression of ROS generation [39], [40]. As such, mouse and human embryonic stem cells have immature mitochondria, reduced expression of OXPHOS enzymes, low metabolic activity, low oxygen consumption, decreased levels of ATP production [41], express modest levels of antioxidant enzymes [40] and have a high glycolytic flux [41]. *In vivo*, embryonic stem cells are maintained in hypoxic conditions (2 to 3%) before vascularization [42] and *in vitro*, low oxygen culture conditions promote maintenance of pluripotency [5]. Rapid changes in ROS production and oxidative status associated with vascularization, growth factor binding, or increased mitochondrial biogenesis and activity, all promote cellular differentiation and determine cell fate [30].

Our findings highlight the capability of NT-plasma to promote osteo and chondrogenesis. The mechanism(s) by which both proliferation and differentiation occur is dependent on both immediate and long-term signaling molecules. In the short term, O_2_^−**.**^ and H_2_O_2_ are produced during the 10 sec NT-plasma exposure, however their sustained increases at 1 and 24 hr after treatment is due to intracellular ROS production. The most likely sources of the increased intracellular ROS are NADPH oxidase and mitochondrial production of O_2_^−**.**^, H_2_O_2_ and NO**^.^** [42], [43]. All three are signaling factors known to participate in cellular proliferation and differentiation by directly activating ROS or RNS-responsive proteins or indirectly by altering the redox status of the cell [44]–[57]. Specifically, MAP kinases are known to respond rapidly to ROS stimulation and these kinases control a wide range of cellular processes including: cellular differentiation, cell cycle control, cytokine and growth factor signaling, survival, hypertrophy and/or apoptosis [14]–[17].

ROS also promote downstream cellular responses by affecting the activity and expression of specific transcription factors [24], [58], [59]. In osteoblast progenitors, continuous production of low levels of H_2_O_2_ stimulates proliferation and augments their potential to differentiate into mature osteoblasts through up-regulation of Runx2 and osterix. [22]. In turn, these transcription factors promote ALKP and osteocalcin expression in differentiating osteoblasts. Similarly, in the current study, NT-plasma induced ROS production increased the expression of Runx2, osterix, ALKP and osteocalcin in pre-osteoblastic MLO-A5 cells.

Conflicting findings have been reported concerning the role of ROS in cell differentiation, but these discrepancies may be due to differing methodologies used for the generation of ROS and the delicate balance required for the appropriate response [57]. In a recent study comparing glucose oxidase continuous production of low levels of H_2_O_2_ to a single bolus of chemical H_2_O_2_, only glucose oxidase generated H_2_O_2_ promoted osteoblast differentiation [22]. This finding is not unexpected as the glucose oxidase system mimics cellular levels of H_2_O_2_ production. In the same way, a 10 sec exposure to NT-plasma initiates low-level intracellular production of ROS. This emphasizes that both timing and ROS concentration are critical in directing cell function. In the same way, chondrocyte differentiation in response to NT-plasma may be directly linked to ROS induced expression of SOX-9 and Runx2, which control collagen and aggrecan expression [60]. In a related study, laser irradiation induced intracellular ROS production and enhanced SOX-9 expression leading to chondrocyte differentiation and expression of collagen and aggrecan [61]. The roles of ROS in progenitor cell proliferation and differentiation described above may also be directly applicable to more complex tissue systems such as regenerating limbs or limb development. While very little has been reported on the direct contribution of ROS in the developmental process, it is well established that a majority of the required signaling molecules (BMPs, WNTs and FGFs) either directly signal through ROS or are indirectly controlled by ROS [62]–[64].

Current studies investigating NT-plasma report multiple effects can be induced in living cells and tissues [5]: these include increased cell proliferation [3], enhanced cell transfection [65]–[67], selective killing of cancer cells [68] and improved wound healing [69], [70]. The multiple effects can be attributed to the *in vitro* conditions selected during cellular treatment with NT-plasma. Results of the current study highlight the use of NT-plasma as a tool to initiate a non-lethal oxidative cellular burst that promotes osteoblast differentiation. Furthermore, these findings suggest that ROS and RNS produced in response to NT-plasma influence signaling pathways that are responsible for cellular proliferation and differentiation. Thus, it would not be unreasonable to assume that NT-plasma could be used to promote musculoskeletal cell differentiation and tissue regeneration.
